# Elevated neutrophil and monocyte counts in peripheral blood are associated with poor survival in patients with metastatic melanoma: a prognostic model

**DOI:** 10.1038/sj.bjc.6602702

**Published:** 2005-07-19

**Authors:** H Schmidt, L Bastholt, P Geertsen, I J Christensen, S Larsen, J Gehl, H von der Maase

**Affiliations:** 1 Department of Oncology, Aarhus University Hospital, Norrebrogade 44, 8000 Aarhus C, Denmark; 2 Department of Oncology, Odense University Hospital, Denmark; 3 Department of Oncology, Herlev Hospital, Hvidovre Hospital, University of Copenhagen, Denmark; 4 Department of Surgical Gastroenterology, Hvidovre Hospital, University of Copenhagen, Denmark

**Keywords:** interleukin-2, prognostic factors, prognostic model, metastatic melanoma, LDH, neutrophils, monocytes

## Abstract

We aimed to create a prognostic model in metastatic melanoma based on independent prognostic factors in 321 patients receiving interleukin-2 (IL-2)-based immunotherapy with a median follow-up time for patients currently alive of 52 months (range 15–189 months). The patients were treated as part of several phase II protocols and the majority received treatment with intermediate dose subcutaneous IL-2 and interferon-*α*. Neutrophil and monocyte counts, lactate dehydrogenase (LDH), number of metastatic sites, location of metastases and performance status were all statistically significant prognostic factors in univariate analyses. Subsequently, a multivariate Cox's regression analysis identified elevated LDH (*P*<0.001, hazard ratio 2.8), elevated neutrophil counts (*P*=0.02, hazard ratio 1.4) and a performance status of 2 (*P*=0.008, hazard ratio 1.6) as independent prognostic factors for poor survival. An elevated monocyte count could replace an elevated neutrophil count. Patients were assigned to one of three risk groups according to the cumulative risk defined as the sum of simplified risk scores of the three independent prognostic factors. Low-, intermediate- and high-risk patients achieved a median survival of 12.6 months (95% confidence interval (CI), 11.4–13.8), 6.0 months (95% CI, 4.8–7.2) and 3.4 months (95% CI, 1.2–5.6), respectively. The low-risk group encompassed the majority of long-term survivors, whereas the patients in the high-risk group with a very poor prognosis should probably not be offered IL-2-based immunotherapy.

The prognosis for patients with metastatic melanoma is poor with a median survival time between 4 and 12 months (Barth *et al*, 1995). Interleukin-2 (IL-2) has resulted in durable responses and cure in 5–7% of the patients (Rosenberg *et al*, 1998; Atkins *et al*, 2000). Meta-analyses on patients receiving IL-2-based immunotherapy have identified independent prognostic factors of survival such as serum lactate dehydrogenase (LDH), number of metastatic sites and performance status (Eton *et al*, 1998; Keilholz *et al*, 1998, 2002; Manola *et al*, 2000). Presently, the strongest prognostic factor in metastatic melanoma is LDH, which is used in the American Joint Committee on Cancer (AJCC) stage IV classification in combination with site of metastases (Balch *et al*, 2001a).

The new paradigm in tumour immunology states that tumour-infiltrating inflammatory cells such as macrophages and neutrophils play a pivotal role in tumour progression and dissemination in a wide range of cancers (Balkwill and Mantovani, 2001; Coussens and Werb, 2002; Pollard, 2004). Recently, baseline elevated neutrophil and monocyte counts in peripheral blood were proposed as prognostic factors for poor survival in patients with metastatic renal cell carcinoma undergoing immunotherapy with IL-2 or *α*-interferon-2b (IFN) (Negrier *et al*, 2002; Atzpodien *et al*, 2003; Donskov *et al*, 2004). Similar data are not available in metastatic melanoma.

We have examined the prognostic impact of neutrophils and monocytes in peripheral blood together with other potentially prognostic factors in 321 patients with metastatic melanoma receiving IL-2-based immunotherapy in consecutive clinical trials between 1990 and 2003. Based on independent prognostic factors, we propose a prognostic model for metastatic melanoma.

## MATERIAL AND METHODS

### Patients

Between April 1990 and December 2003, 321 patients with metastatic melanoma were entered on consecutive clinical trials with IL-2-based immunotherapy. Data were analysed per 1 February 2005, that is, more than 1 year after the last patient had received IL-2-based immunotherapy. The main inclusion criteria consisted of biopsy-verified stage IV disease, a WHO performance status of 2 or better and age above 18 years. The main exclusion criteria were brain metastases, a previous malignant disease other than nonmelanoma skin cancer, symptomatic heart or lung disease, seizure disorders, serious autoimmune disorders, concomitant corticosteroid therapy or previous systemic treatment for metastatic disease. The ethics committees at the involved counties approved the present project, and written informed consent was obtained from each patient. Data were collected from the three participating institutions, and all cases were reviewed individually and entered in a central database. The data set was complete with the exception of one missing monocyte count from a single patient.

### Treatments

In all, 20 patients received recombinant IL-2 and IFN. Interleukin-2 was administered at a dose of 18 MU m^−2^ day^−1^ by a 24-h continuous intravenous (i.v.) infusion. Interferon was administered during the IL-2 infusion periods at a dose of 3 MU m^−2^ day^−1^. The treatment plan consisted of two induction cycles and four maintenance cycles with a 3-week rest period following each cycle. Each induction cycle consisted of two IL-2 infusion periods of 120 and 108 h duration, respectively, separated by 6 days rest period. Each maintenance cycle consisted of a 120 h IL-2 infusion period.

In all, 86 patients received IL-2, IFN and cisplatinum (Schmidt *et al*, 2000). One cycle consisted of cisplatinum i.v. bolus on day 1 at a dose of 100 mg m^−2^. Recombinant IL-2 was administered subcutaneous (s.c.) at a dose of 9 MU m^−2^ twice daily on days 5–9 and at a dose of 4.5 MU m^−2^ daily on days 12, 14 and 16. Interferon was administered s.c. at a dose of 10 MU daily on days 2–4 and thereafter three times weekly.

In total, 83 patients received IL-2, IFN±histamine (Schmidt *et al*, 2002). One cycle consisted of IL-2 s.c. at a dose of 9 MU twice daily on days 4–8, and daily on days 11–15. Patients received INF at a dose of 5 MU s.c. daily from days 1 to 21. Histamine dihydrochloride was administered s.c. at a dose of 1 mg over 20 min, twice daily. Histamine injections were administered from day 4 and on the same days as IL-2 and INF injections.

A total of 102 patients received IL-2 s.c.±low-dose total body irradiation (LTBI). One cycle consisted of IL-2 s.c. on days 2–5 at a dose of 18 MU twice daily, and at a dose of 9 MU twice daily on days 9–12. A single fraction of LTBI 0.1 Gy were given on days 1 and 8.

In all, 30 patients received intratumoral injections of bleomycin and electroporation (electrochemotherapy) followed by IL-2 s.c. at a dose of 2 MU daily for 21 consecutive days (Gehl *et al*, 2003).

### Statistics

Calculations were performed using SPSS (version 11.5) statistical software. Univariate and multivariate Cox's analyses were performed to investigate the prognostic impact of baseline factors in relation to survival. Factors included were WHO performance status (0–1 *vs* 2); number of metastatic sites (1–2 *vs* ⩾3); site of metastases (skin and lymph nodes *vs* lungs *vs* other visceral sites); baseline serum LDH (normal *vs* elevated); baseline blood neutrophil counts (normal *vs* elevated); and baseline blood monocyte counts (normal *vs* elevated). Baseline blood measurements (LDH, neutrophils and monocytes) were both analysed as continuous variables (absolute values) and as dichotomous variables using the upper normal reference level from each of laboratories in the participating institutions. Survival was calculated from the day of treatment start to the end point (death or censoring). Patient survival and median duration of response was analysed by the Kaplan–Meier method. The simultaneous relationship of multiple prognostic factors for survival was assessed using Cox's proportional-hazards model. Factors with a *P*-value <0.10 in the univariate analyses were included in the multivariate analysis to identify factors of independent significance. The multivariate analysis was stratified according to treatment regimens. The multivariate model selection was performed by a stepwise strategy using the likelihood ratio test to create a multiple risk factor model. Hazard ratios were calculated to estimate the magnitude and the direction of the effect. Schoenfeld residuals and log(−log(S)) *vs t* plots were evaluated to assure the assumption of proportional hazards. Assessment of the model was carried out using crossvalidation techniques (Altman and Royston, 2000), using SAS (version 8.2) statistical software. All survival data were updated on 1 February 2005.

## RESULTS

The median survival was 8.1 months (range 1–188), and 19 of 321 patients were currently alive. Of these 19 patients, 17 had a survival length of more than 24 months and were termed long-term survivors. Data were analysed more than 1 year after the last patient had received IL-2-based immunotherapy and the median follow-up time of the 19 patients currently alive was 52 months (range 15–188 months).

### Univariate analyses of pretreatment variables

**Figure 1:**
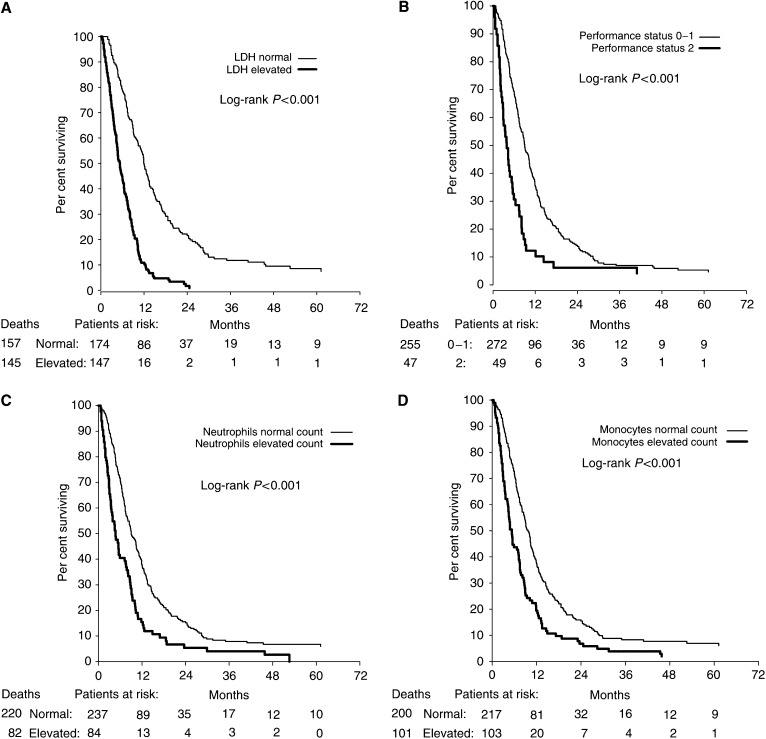
Kaplan–Meier survival estimates for 321 patients with metastatic melanoma according to baseline: (**A**) lactate dehydrogenase (LDH), (**B**) performance status, (**C**) blood neutrophils and (**D**) blood monocytes.

In univariate analyses, the following baseline factors were statistically significantly (*P*<0.05) associated with poor survival: elevated LDH (*P*<0.001), a performance status of 2 (*P*<0.001), elevated neutrophil counts (*P*<0.001), elevated monocyte counts (*P*<0.001) (Figure 1A–D), more than two metastatic sites (*P*<0.001) and site of metastases (*P*=0.03) (Table 1). Elevated LDH, neutrophil and monocyte counts refer to levels above the upper normal reference levels.

### Multivariate analyses of pretreatment variables

Upon entering the significant variables from the univariate analyses into a multivariate Cox's analysis, three variables turned out as independent factors of poor survival: elevated LDH (*P*<0.001), elevated neutrophil counts (*P*=0.02) and a performance status of 2 (*P*=0.008) (Table 2). Similar results were achieved when entering blood counts as absolute values (data not shown). In patients with a normal LDH, the 5-year survival rate was 9% compared to 0% in patients with an elevated LDH (Figure 1A). Similarly, in patients with a normal neutrophil count, the 5-year survival rate was 7% compared to 0% in patients with an elevated count (Figure 1C). Model assessment using crossvalidation revealed that the model was robust, and there was little evidence of overfitting. The alternative multivariate model including monocytes, instead of neutrophils, yielded a similar result (likelihood ratio *χ*^2^ of 87.7 *vs* 86.6).

### Prognostic model

**Figure 2:**
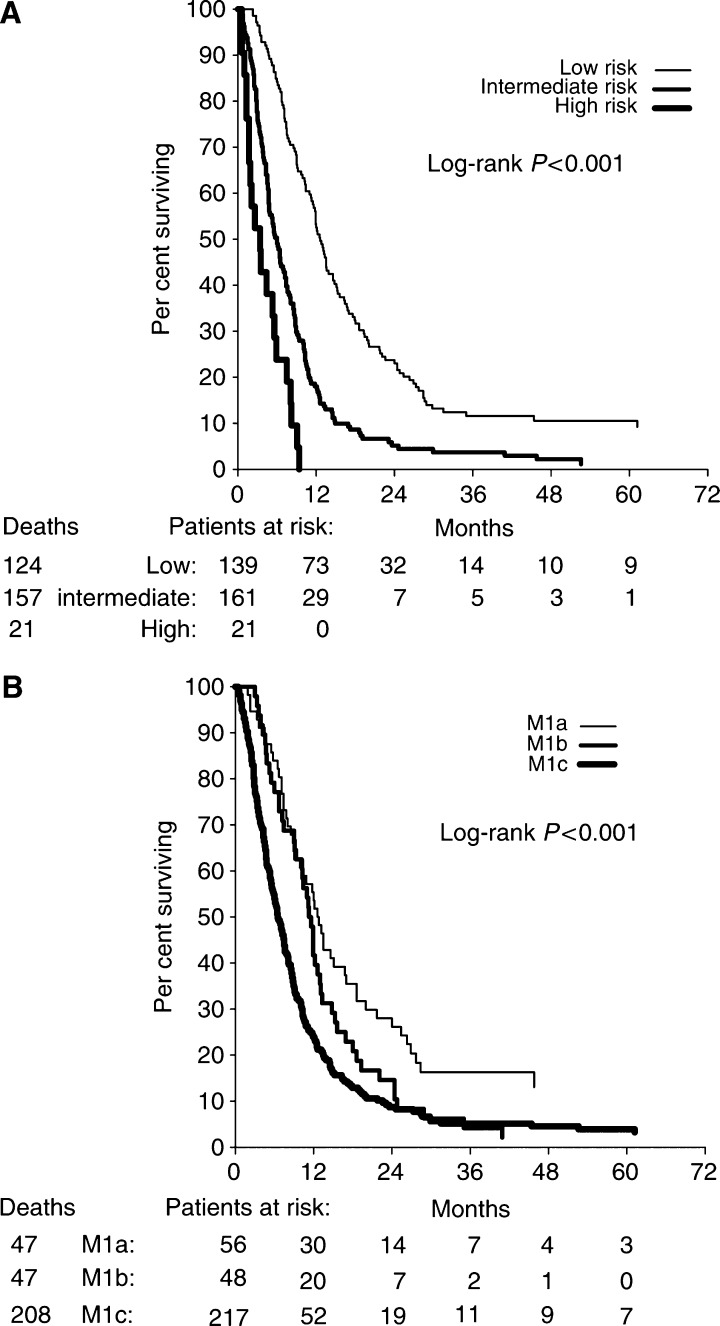
Kaplan–Meier survival estimates for 321 patients with metastatic melanoma according to combination factors: (**A**) prognostic model with low, intermediate and high risk. (**B**) AJCC stage IV classification: M1a, normal lactate dehydrogenase (LDH) and metastases confined to the skin and lymph nodes, M1b including lung metastases and normal LDH and M1c including other visceral organs or elevated serum LDH.

Based on the ratios of regression coefficients (log hazard ratios in the final Cox's model) of variables, we defined the weights of prognostic factors as follows: elevated LDH was assigned weight 2, elevated neutrophil counts weight 1 and performance status of 2 weight 1. A prognostic score of the cumulated weights of these variables was used to assign patients to low-risk (none elevated, score 0), intermediate-risk (any combination of 1–2 elevated variables, score 1–3) and high-risk (all three variables elevated, score 4) groups, respectively. The median survival of low-risk (*n*=139), intermediate-risk (*n*=161) and high-risk patients (*n*=21) was 12.6 months (95% confidence interval (CI) 11.4–13.8), 6.0 months (95% CI, 4.8–7.2) and 3.4 months (95% CI, 1.2–5.6), respectively (Figure 2A). The 5-year survival rates for these three groups were 9, 1 and 0%, respectively. The predicted 12-month survival probabilities were similar to the respective Kaplan–Meier estimates for each risk score. The 12-month survival probabilities predicted by the Cox's model were 48% (low risk), 14% (intermediate risk) and 1% (high risk), and the Kaplan–Meier estimates were 51, 13 and 0%, respectively. Similar differences were observed for the 24-month survival probabilities.

We compared our proposed prognostic model with the current AJCC classification of stage IV disease, which includes LDH, and also the site of metastases. The AJCC model defines stage M1a as skin or lymph node metastases with a normal LDH, M1b as lung metastases with a normal LDH and M1c as other visceral metastases or an elevated LDH. In the univariate analyses, we found a significant difference in survival time between stage M1a and M1c (*P*<0.001), but not between M1a and M1b (*P*=0.11) (Figure 2B). When entering the AJCC model together with our proposed model into a multivariate analysis, the prognostic index of LDH, neutrophils and performance status remained highly statistically significant through all levels (*P*<0.001), while the AJCC classification (*P*=0.26) had no independent prognostic impact (Table 3).

## DISCUSSION

Recent studies of IL-2-based immunotherapy in metastatic melanoma have identified mainly LDH, performance status, number of metastatic sites and sites of metastases as independent prognostic factors (Sirott *et al*, 1993; Eton *et al*, 1998; Keilholz *et al*, 1998, 2002; Manola *et al*, 2000). Our findings of LDH and performance status as independent prognostic factors were therefore expected. However, the prognostic impact of neutrophils and monocytes in peripheral blood was a novel finding. Both elevated neutrophil and monocyte counts were associated with poor survival in the univariate analyses, and if neutrophils were forced out of the multivariate model, monocytes became an independent prognostic factor together with LDH and performance status. The neutrophil and monocyte counts were highly significantly correlated in the patient material (Spearman's rank-correlation coefficient 0.49, *P*<0.0001), which explains why only one of them was an independent factor in the multivariate analysis. Thus, the neutrophil count could be replaced by the monocyte count resulting in a similar prognostic score.

Our findings in patients with metastatic melanoma correspond with observations in metastatic renal cell carcinoma. Thus, an elevated neutrophil count was an independent risk factor for poor survival in two large studies in metastatic renal cell carcinoma (Negrier *et al*, 2002; Atzpodien *et al*, 2003), whereas the monocyte count was not considered in these studies. In a third study, both elevated neutrophil and elevated monocyte counts were statistically significant correlated to poor survival however, in univariate analyses (Donskov *et al*, 2004). The explanation for the association between high neutrophil or monocyte counts and poor prognosis is not fully clarified. The new paradigm in tumour immunology states that the tumour microenvironment can educate and control invading leucocytes to promote angiogenesis, viability, motility and invasion (Hanahan and Weinberg, 2000; Balkwill and Mantovani, 2001; Coussens and Werb, 2002; Lin and Pollard, 2004). Especially tumour-associated macrophages, which arise from blood monocytes, seem to play a crucial role in this interaction (Pollard, 2004). The chemokines CXCL1 (Gro-*α*) and CXCL8 (IL-8) are constitutively produced by melanoma cells and the corresponding receptors CXCR1 and CXCR2 are expressed on melanoma cells as well as on macrophages, neutrophils and eosinophils (Moser *et al*, 1993; Norgauer *et al*, 1996; Dhawan and Richmond, 2002). The autocrine production of these chemokines by melanoma cells increases their survival, proliferation and dissemination, as well as attracts inflammatory cells, such as monocytes and neutrophils (Haghnegahdar *et al*, 2000; Schaider *et al*, 2003; Balkwill, 2004). These tumour-infiltrating neutrophils can produce VEGF, IL-8 and matrix metalloproteinases involved in tumour invasion and angiogenesis (Shamamian *et al*, 2001; Schaider *et al*, 2003). In one study, IL-8 was detectable in the serum in 50% of patients with metastatic melanoma, which correlated with tumour load (Scheibenbogen *et al*, 1995). These findings indicate a link between elevated neutrophils and monocytes in peripheral blood and especially aggressive melanomas leading to a poor prognosis.

The high-risk group in our proposed prognostic index with elevated neutrophil counts, elevated LDH and a performance status of 2, had a very poor prognosis with a median survival of only 3.4 months (95% CI, 1.2–5.6). None of these patients survived for more than 10 months, and this group of patients should probably not be offered IL-2-based immunotherapy. In contrast, the low-risk group with no elevated risk factors had a median survival of 12.6 months (95% CI, 11.4–13.8) and included 15 of the 17 long-term survivors. It should be emphasised that the majority of patients in our study were treated with intermediate s.c. IL-2 regimens and these results cannot directly be applied to patients treated with high-dose i.v. IL-2. However, our hypothesis is that the findings will be similar for such a group of patients.

We applied our data to the AJCC stage IV classification (version 2001), and observed a significant difference in survival between patients with skin/lymph node metastases (M1a) and visceral metastases or an elevated LDH (M1c), but not between M1a and patients with lung metastases (M1b). These results correspond well with the data reported by Balch *et al* (2001b) demonstrating a significant survival difference between M1a and M1b at 1 year, but not beyond that time frame. When we tested our prognostic model against the AJCC model in a multivariate analysis, the prognostic impact of our model based on neutrophils, LDH and performance status (*P*<0.001) was superior compared to the AJCC model (*P*=0.22). Similarly, Keilholz *et al* (2002) have reported that the prognostic impact of a combination of LDH and performance status was superior to the AJCC model. Our results further supplements that model by adding neutrophil counts. The proposed model has been internally validated (Altman and Royston, 2000), but validation in an independent study is obviously warranted.

In conclusion, the independent prognostic impact of elevated neutrophil or monocyte counts in peripheral blood is a novel finding in patients with metastatic melanoma. As expected, LDH and performance status were also independent prognostic factors. Our proposed prognostic model with neutrophil counts, LDH and performance status was able to identify a low-risk group encompassing the majority of long-term survivors, and a high-risk group with a very poor prognosis, which should probably not be offered IL-2-based immunotherapy. The validation of the prognostic model in an independent study is warranted.

## Figures and Tables

**Table 1:** Univariate Cox's analyses of risk factors and survival in metastatic melanoma

			**Univariate Cox's analyses**		
**Risk factors**	**Categories compared**	**Number of patients**	**Hazard ratio**	**95% CI**	***P*-value**
Gender	Female *vs* male	181 *vs* 140	1.0	0.8–1.3	0.88
Age (median 51 years)	<51 *vs* ⩾51	157 *vs* 164	1.1	0.9–1.3	0.59
Performance status	0–1 *vs* 2	272 *vs* 49	2.1	1.5–2.8	<0.001
No. of metastatic sites	1–2 *vs* ⩾3	195 *vs* 126	1.6	1.3–2.0	<0.001
Location of metastases	Skin, lymph nodes *vs* lung *vs* visceral sites	82 *vs* 72 *vs* 167			0.03*
	Skin, lymph nodes *vs* lung	82 *vs* 72	1.3	0.9–1.8	0.11
	Skin, lymph nodes *vs* visceral sites	82 *vs* 167	1.5	1.1–1.9	0.01
LDH	Normal *vs* elevated	174 *vs* 147	2.9	2.3–3.7	<0.001
Neutrophil counts	Normal *vs* elevated	237 *vs* 84	1.9	1.4–2.4	<0.001
Monocyte counts	Normal *vs* elevated	217 *vs* 103	1.7	1.4–2.2	<0.001

CI=confidence interval; LDH=lactate dehydrogenase.

* *P*-value is an overall estimate for all levels.

**Table 2:** Multivariate Cox's model of independent prognostic factors for survival in metastatic melanoma

		**Multivariate Cox's analysis^a^**				
**Risk factors**	**Categories compared**	**Hazard ratio**	**95% CI**	***P*-value**	**Regression coefficients**	**Weight (contribution to cumulative risk score)**
Performance status	0–1 *vs* 2	1.6	1.1–2.3	0.008	0.47	0 *vs* 1
LDH	Normal *vs* elevated	2.8	2.2–3.6	<0.001	1.03	0 *vs* 2
Neutrophils	Normal *vs* elevated	1.4	1.1–1.8	0.02	0.32	0 *vs* 1

CI=confidence interval; LDH=lactate dehydrogenase. All other variables (location of metastases, number of metastatic sites and blood monocytes) were not significant and therefore excluded from the model.

a In all, 320 patients and 301 deaths. Analysis stratified by treatment regimen.

**Table 3:** Univariate and multivariate Cox's analyses of the proposed prognostic index and the AJCC stage IV classification in patients with metastatic melanoma

			**Univariate analyses**			**Multivariate analysis^a^**		
**Combination of risk factors**	**Categories compared**	**Number of patients**	**Hazard ratio**	**95% CI**	***P*-value**	**Hazard ratio**	**95% CI**	***P*-value**
LDH, neutrophils and performance status	Low *vs* intermediate *vs* high	139			<0.001*			<0.001*
	Low *vs* intermediate	161	2.4	1.9–3.0	<0.001	2.2	1.7–2.9	<0.001
	Low *vs* high	21	5.9	3.7–9.6	<0.001	5.4	3.3–9.0	<0.001
								
AJCC stage IV classification	M1a *vs* M1b *vs* M1c	56			<0.001*			0.26*
	M1a *vs* M1b	48	1.4	0.9–2.1	0.11	1.3	0.9–2.0	0.21
	M1a *vs* M1c	217	2.0	1.5–2.8	<0.001	1.3	0.9–1.8	0.11

CI=confidence interval; LDH=lactate dehydrogenase; AJCC=American Joint Committee on Cancer; M1a=skin and lymph node involvement with normal serum LDH; M1b=including lung metastases with normal serum LDH; M1c=other visceral metastases or an elevated serum LDH. Low risk denotes a normal LDH, and a normal neutrophil count, and a performance status of 0–1. Intermediate risk denotes any combination of at least one but not all variables elevated. High risk denotes an elevated LDH, an elevated neutrophil count, and a performance status of 2.

* *P*-values are overall estimates for all levels.

a *N*=321, 302 deaths.
